# Calf health from birth to weaning - an update

**DOI:** 10.1186/s13620-021-00185-3

**Published:** 2021-03-16

**Authors:** Ingrid Lorenz

**Affiliations:** Bavarian Animal Health Service, Poing, Germany

**Keywords:** Calf health, Calf welfare, Disease prevention, Colostrum management, Dairy calf nutrition, Cow‐calf separation, Calf housing

## Abstract

Research on calf health and welfare has intensified in the past decades. This is an update on a review series on calf health from birth to weaning published ten years ago.

Good colostrum management is still recognised as the single most important factor to preventing calf morbidity and mortality, however, it is now known that immunoglobulins are only one of many components of colostrum that are vital for the calf’s development. Other non-nutrient factors like leucocytes, hormones and growth factors, oligosaccharides as well as microRNAs have significant effects on the development and maturation of the intestinal and systemic immune functions. They also promote the maturation and function of the intestine, thus enabling the calf to digest and absorb the nutrients provided with colostrum and milk. The improved energetic status of colostrum-fed neonates is reflected by an accelerated maturation of the somatotropic axis, which stimulates body growth and organ development. Colostrum oligosaccharides are presumed to play a major role in the development of a healthy intestinal flora.

A biologically normal (intensive) milk-feeding programme is subsequently necessary for optimal body growth, organ development and resistance to infectious diseases. Ad-libitum or close to ad-libitum feeding in the first three to four weeks of life also leaves calves less hungry thus improving calf welfare. Only calves fed intensively with colostrum and milk are able to reach their full potential for performance throughout their life.

Public interest in farm animal welfare is growing in past decades, which makes it necessary to have a closer look at contentious management practices in the dairy industry like early separation of the dairy calf from the dam with subsequent individual housing. Public objection to these practices cannot be mitigated through educational efforts. Contrary to common opinion there is no evidence that early cow-calf separation is beneficial for the health of calf or cow. There is evidence of behavioural and developmental harm associated with individual housing in dairy calves, social housing improves feed intake and weight gains, and health risks associated with grouping can be mitigated with appropriate management.

In conclusion, there are still many management practices commonly applied, especially in the dairy industry, which are detrimental to health and welfare of calves.

## Introduction

Calfhood diseases have a major impact on the economic viability of cattle operations, due to the direct costs of calf losses and treatment and the long term effects on performance [1]. After calf health was prioritised as one of the most important animal health issues facing the Irish livestock industry in an expert Policy Delphi study conducted on behalf of Animal Health Ireland (AHI) [2] in 2010, all aspects of calf health from birth to weaning have been reviewed [3–5] as a scientific basis for providing evidence-based information on successful calf rearing to Irish Farmers. An update of these reviews was made necessary by a multitude of new scientific findings, especially in the area of colostrum management, further feeding of the dairy calf and other calf welfare related topics. This article considers the most important scientific evidence since 2011 that either substantiates or changes our understanding of calf health from birth to weaning.

## Colostrum management

Good colostrum management is widely recognised as the single most important factor to preventing calf morbidity and mortality [6]. Traditionally most of the positive effects of colostrum are attributed to the high content of immunoglobulins (Ig). Calves are indeed born without protective immunoglobulins (Ig) due to the structure of the bovine placenta and therefore depend on the successful passive transfer of maternal Ig from colostrum [7]. However, colostrum provides a variety of other important ingredients. In the past years the knowledge about the biological significance of these ingredients increased considerably.

### Important non-nutrient factors in colostrum

Bovine colostrum contains high concentrations of viable maternal leucocytes similar to those in peripheral blood, but with relatively more macrophages/monocytes and fewer lymphocytes [8]. Maganck et al. [9] identified the lymphocytes mainly as T-lymphocytes. The uptake of colostral leucocytes through the intestinal barrier is possible with the preferential route of uptake being through follicle-associated epithelium of Peyer’s patches [8]. Viable maternal leucocytes are destroyed by freezing [10] and significantly reduced by heat treatment [11]. Studies comparing the effects of cell-free (through lysis of cells by flash-freezing in liquid nitrogen) vs. whole colostrum in calves found significant differences in number and composition of blood monocytes [12] and lower numbers of B- and T- cells at some timepoints after vaccination in calves receiving cell-free colostrum [13]. However, the effect of maternal leucocytes on common calfhood diseases has so far been found to be small, with only one out of three studies finding a significantly higher chance of antibiotic treatment and a higher frequency of fever in calves that received cell-free colostrum [12, 14, 15].

Bovine colostrum contains bioactive proteins in higher abundance then mature milk. When the proteomes of colostrum and milk are categorized according to their biological function, the largest difference is observed in the category of proteins responsible for metabolic processes [16]. In particular, colostrum contains a large number of growth factors, including insulin-like growth factors (IGFs) and insulin in high concentrations [17]. These substances mainly exert their effects locally, since receptors for IGF-I, IGF-II and insulin can be found throughout the intestines in newborn calves, however, absorption is negligible [18]. While IGF-I alone does not influence development of the intestinal epithelium in an experimental setting, an extract of first-milked bovine colostrum increased villus circumferences and heights in small intestine, as well as epithelial cell proliferation rate in the intestines. The mechanism responsible for this observation is not clear [19]. Feeding of colostrum as opposed to formula with comparable nutrient content stimulates mucosal growth and increases the absorptive capacity in the small intestines [20, 21]. Increased nutrient and glucose absorption thus allows increased glucose supply and hepatic glycogen storage, which improves the glucose status. The improved energetic status of colostrum-fed neonates is reflected by an accelerated maturation of the somatotropic axis, leading especially to enhanced production of IGF-I in the neonate and thus promoting anabolic processes. [22].

Another substance group present in bovine colostrum in high concentrations are oligosaccharides [23], whose effects are not yet completely understood. However, they are very likely to prevent pathogen adhesion to the intestinal epithelium [24], may enhance the uptake of IgG [25], and serve as carbon sources for beneficial bacteria to promote their growth and establishment in the intestine [26].

Colostrum also contains high amounts of microRNAs. MicroRNAs are short, non-coding RNA molecules that can regulate gene expression at the post-transcriptional level and which are believed to provide a method of signalling from the mother to the neonate. They can reach the intestines and be absorbed into the bloodstream since they are sheltered from digestion in extracellular vesicles. MicroRNAs may play an important role in the development of the intestinal epithelium, as well as the maturation of the calves immune system [27].

### Extended feeding of colostrum

Extended feeding of colostrum or a mixture of colostrum and milk for three days not only increases Ig concentration in the serum [28], but also promotes small intestinal growth and maturation [29]. Feeding transition milk decreases the incidence of calves suffering from disease [30]. In conclusion, it can be assumed that bioactive substances from colostrum other than Ig highly effect intestinal health in the neonatal calf through enhancing gut maturation and immunity [18].

### Testing of colostrum quality

However, passive transfer of Ig from the dam to the calf via colostrum still plays the major role in providing immediate systemic immunity after the calf is born. Colostrum quality is defined by Ig concentration and remains one crucial factor for successful passive transfer. Since direct laboratory methods for the determination of Ig in colostrum are elaborate and expensive, the use of cow-side testing of colostrum quality has been intensively investigated. Buczinski and Vandeweerd [31] conducted a systematic review and meta- analysis on the diagnostic accuracy of Brix refractometry for the assessment of bovine colostrum quality. Evaluation of 11 studies representing 4,251 colostrum samples showed that a cut-point of 22 % Brix can be used to diagnose good quality colostrum (IgG ≥ 50 g/L) with a post-test probability of 94.3 % (90.7–96.9 %), whereas the posttest probability of good colostrum with a Brix value < 18 % was only 22.7 % (12.3–39.2 %). The authors suggest using the two cut-points to select good quality colostrum (sample with Brix ≥ 22 %) or to discard poor quality colostrum (sample with Brix < 18 %). When sample results are between these two values, supplementation with higher quality colostrum should be considered.

### Monitoring of passive immunity status

For many decades an individual calf threshold of 10 g/L of serum Ig has been used to define failure of passive transfer of immunity. A serum protein concentration of 52 g/L was found to be equivalent to 10 g/L serum IgG and was suggested as test threshold for healthy calves up to an age of 8 days [32]. Recent studies challenged this simplistic approach to the monitoring of passive immunity status. Windeyer et al. [33] found a decreased risk for respiratory disease in calves with serum total protein values ≥ 57 g/L, Chigerwe et al. [34] considered serum total protein levels between 58 and 63 g/L as optimum for indicating adequate passive transfer of immunity in dairy calves. The USDA National Animal Health Monitoring System’s Dairy Study 2014 [35] showed, that the risk for calf morbidity and mortality decreased significantly with increased serum Ig concentrations. In light of these findings, a panel of calf specialists revised the standards for passive immunity transfer for the US dairy industry according to latest scientific evidence [36]. The proposed standard includes 4 serum IgG categories: excellent, good, fair, and poor with serum IgG levels of ≥ 25.0, 18.0–24.9, 10.0–17.9, and < 10 g/L, respectively. Table 1 shows the corresponding total protein and Brix % levels, as well as suggested achievable standards for the four categories on a herd level.

**Table 1:** Consensus serum IgG concentrations and equivalent total protein (TP) and Brix measurements, and percentage of calves recommended in each transfer of passive immunity category (adapted from Lombard et al. [36])

Passive transfer of immunity category	Serum IgG category (g/L)	Equivalent serum total protein (g/L)	Equivalent serum Brix %	Recommended percentage of calves on farm in category
Excellent	≥ 25.0	≥ 62	≥ 9.4	> 40
Good	18.0–24.9	58–61	8.9–9.3	~ 30
Fair	10.0–17.9	51–57	8.1–8.8	~ 20
Poor	< 10.0	< 51	< 8.1	< 10

### Hygienic quality of colostrum

Bacterial contamination of colostrum occurs frequently on many dairies, with two associated concerns; a risk of transfer of infection and decreased absorption of IgG in the intestines. Total bacterial count should not exceed 100,000 cfu/mL, and faecal coliforms should be below 10,000 cfu/mL [37]. Heat treatment of colostrum without reducing IgG levels and changing viscosity is possible at 60 °C for 60 min [38]. However, even though IgG levels remain stable and the bacterial count can be largely reduced, heat treatment of colostrum also reduces the content of somatic cells, IgA, insulin, IGF-I, as well as other proteins, which could result in biologically important effects on the developing immune system of the neonate fed heat-treated colostrum [11].

## Further nutrition and weaning of the dairy calf

For most of the last century research in the area of calf nutrition and management focused on reducing the amounts of whole milk fed to dairy calves prior to weaning by all means possible [39]. This led to the traditional recommendation to feed dairy calves milk or milk replacer to an amount of approximately 10 % of the their body weight (BW) per day [40]. This concept of “restricted feeding” was introduced to encourage calves to eat concentrates as early as possible and thus to minimise costs for relatively expensive liquid feeds. However, starter concentrate intake is negligible in the first 3 weeks of life, irrespective of the amount of milk fed [41, 42]. After the first 3 weeks of life, starter concentrate intake increases which allows for compensatory growth [40]. This feeding regime only allows for maintenance requirements and minimal weight gain in early calfhood under thermo-neutral and otherwise favourable conditions [43]. It has been known for a long time that calves can grow a lot faster if they are supplied with more nutrients [44, 45]. However, worldwide interest in early calf nutrition has only been increasing since the beginning of the current century. Diaz et al. [46] showed in a study published 2001 that the recommendations for nutrient requirements of the young calf were inadequate at the time. By feeding three different levels of milk replacer with high protein content they also demonstrated that calves on a higher plain of nutrition did not only gain weight faster, but also showed higher feed efficiency. Protein deposition increased linearly with feeding rate, whereas fat deposition increased less from the middle to highest feeding rate, indicating that on higher plains of nutrition a high protein content of the milk replacer supports lean tissue growth. Furthermore, early studies demonstrated that calves suckling their dam or otherwise fed ad libitum ingest about 20 % of body weight (BW) per day in milk and can reach more than one kg of daily weight gain [41, 47]. Feeding milk ad-libitum or close to ad-libitum will delay starter intake, which has to be taken into account for the weaning process [48]. In two seminal studies Kahn et al. [42, 49] presented a method which allows feeding high volumes of milk in the first three to four weeks of live without the drawback of stagnating growth during weaning due to poor starter intake. They compared calves on conventional restricted feeding (10 % of body weight) with calves that received close to ad-libitum volumes of milk (20 % of body weight) for 23 and 25 days, respectively. Subsequently milk volumes were gradually reduced to 10 % of body weight over four days (step-down) in calves previously fed close to ad-libitum. Both groups were weaned on day 50. Calves in the step-down group did not only gain weight and grow much faster than conventionally fed calves, they also showed higher starter and forage intake after the reduction of feeding volumes. This also resulted in a higher forestomach weight, ruminal wall thickness and better papillae development [42]. In one study conventionally fed calves showed significantly more days with diarrhoea in the third and fourth week of live than step-down calves [49]. A different approach to avoid growth check during weaning of ad-libitum fed calves is to delay weaning age up to the twelfth week of live [48].

Meanwhile numerous studies identified the effects of biologically normal milk feeding programs (also called intensified or accelerated feeding programs) when compared with conventional restricted feeding. The importance of the feeding intensity on gastrointestinal and systemic development of calves has recently been reviewed [18]. Besides the obvious increased body weight and body growth, enhanced organ growth and development (e.g. rumen, small intestine, mammary gland) as well as stimulation of the endocrine pancreas have been found. Metabolic changes include greater systemic metabolic activity, elevated metabolic activity in the ruminal epithelium and in the omental adipose tissue. Analogous to the effect of colostrum, a high plain of milk feeding is necessary to stimulate the somatotropic axis and to enhance maturation of the intestinal immune system [18].

The influence of early calf nutrition on lifetime performance was first examined by Soberon et al. [50]. Analysing data from two dairy herds they found a significant correlation between preweaning average daily gain (ADG) and milk yield in first lactation. For every 1 kg of preweaning ADG, heifers in the two herds produced 850 kg and 1113 kg more milk during their first lactation, respectively. The authors concluded that early life programming for lifetime performance must commence immediately after birth in form of liquid feed. Soberon and Van Amburgh [51] conducted a meta-analysis of 12 data sets that reported early-life intake and growth rates as well as first-lactation milk production. In that study calves fed more milk or milk replacer in early life were two times more likely to have a greater milk yield in first lactation and for every kilogram of preweaning ADG, first lactation milk yield increased by 1,550 kg. A second meta-analysis concluded that preweaning ADG minimally affects milk production when growth rate is below 0.5 kg/d, but has a greater influence as growth rate increased from 0.5 to 0.9 kg/d, indicating that the long term benefits from accelerated growth in calves can only be gained if early calf nutrition allows for close to normal growth rates [52].

Restricted feeding of dairy calves also has animal welfare implications. Calves fed limited amounts of milk show behaviours indicative of chronic hunger. Calves provided 5 L/d of milk vocalized at higher rates and with higher pitch calls compared with calves fed 8 L/d [53]. If calves are fed by automatic milk feeding systems the number of unrewarded visits (i.e., visits to the feeder when calves are not entitled to receive milk) increases markedly with decreasing milk allowance [54–58]. Calves fed ad-libitum performed on average 2.1 unrewarded visits per day [57], whereas restrictively fed calves entered the feeder up to 35 times per day without being entitled to receive milk [55]. Restricted milk allowance also reduces play behaviour [59], which can also serve as an indicator of animal welfare [60].

## Feeding of colostrum and milk containing residues of antimicrobial substances

Feeding of waste milk (milk unfit for human consumption) potentially containing residues of antimicrobial substances is common practice in the dairy industry worldwide [61–65]. In the European Union feeding of milk containing residues of antimicrobials to calves on the farm of origin is not generally prohibited [66]. The exposure of microorganisms to subinhibitory concentrations of antimicrobials is known to select for antimicrobial resistance [67]. Several studies reported the shedding of an increased proportion of antimicrobial-resistant faecal bacteria when calves are fed waste milk containing antimicrobial residues [68–70]. Pereira et al. [71] fed milk experimentally spiked with ceftiofur, penicillin, ampicillin, and oxytetracycline below the minimum inhibitory concentrations and found a significantly greater proportion of E. coli resistant to ampicillin, cefoxitin, ceftiofur, streptomycin and tetracycline when compared to calves fed with antimicrobial-free milk. Also, a greater proportion of *E. coli* isolates from calves fed residue containing milk were resistant to 3 or more antimicrobial drugs when compared to the control group.

The reason for feeding waste milk containing milk under withdrawal from cows treated with antibiotics during lactation is mainly economical in nature [62]. This differs from the feeding of colostrum potentially containing antimicrobial residues due to dry cow treatment (DCT), since the adequate provision of colostrum is of major importance for the survival, health and performance of the calf as detailed previously. A scientific opinion by the European Food Safety Authority (EFSA) Panel on Biological Hazards (BIOHAZ) [66] comes to the conclusion that faecal shedding of antimicrobial-resistant bacteria will not increase when calves are fed colostrum from cows undergoing DCT if the interval from the dry‐off treatment until calving is as long as or longer than the minimum specified in the Summary of Product Characteristics of the antimicrobial product. However, this finding is based on limited scientific evidence and in one field study on antimicrobial residues in colostrum, one third of the samples showed residues in concentrations above maximum residue limits (MRL), mostly cloxacillin [66]. Another study analysing colostrum samples of 200 cows that underwent DCT for the presence of β-lactam antimicrobials found residues of cloxacillin in 49 colostrum samples (in 34 samples with concentrations above the MRL) from 88 cows that were treated with cloxacillin- containing products. However, looking into the different commercial products used, the vast majority of positive samples were obtained from cows treated with one formulation providing an extended period of protective antibiotic levels in the udder. Residues of other β-lactam antimicrobials were rarely found [72].

There are initial reports indicating that feeding waste milk containing antimicrobial residues does not only foster selection of antibiotic resistances but also has an impact on the composition of the intestinal [73–75] and nasal [75] microbiota.

## Early separation from the dam and individual housing of the dairy calf

Under natural conditions, cows separate from the herd before giving birth and subsequently keep their calf away from the herd for some days. After that, calves join the herd to form groups with their peers engaging in social relationships [76].

On the vast majority of commercial dairy farms, however, calves are separated from their dams within hours or at least within very few days after they are born. There are several perceived reasons for this practice by producers, as summarized by Flower and Weary [76]. First of all, there are economic reasons, since calves left with cows to suckle would consume high volumes of saleable milk, whereas handfed calves are often fed on waste milk or less expensive milk replacer. Suckling is thought to interfere with return to oestrus and with milk led-down in cows. Furthermore, handfed calves are easier to monitor for colostrum intake and general health. Finally, there is a compassionate argument that prolonged time to bond between cow and calf would increase separation distress. Veterinarians consider improved calf health to be the main reason to support the early separation of cow and calf [77].

On the other hand, public interest in the welfare of farm animals is rising, increasing the potential for contentious practices to damage the public image of the industry. When asked “Should dairy calves be separated from the cow within the first few hours after birth?” 76 % of those without involvement in the dairy industry objected to the practice [78]. In an online study including German and US citizens, the majority of participants were in favour of keeping cows and calves together for a prolonged period of time [79]. Ventura et al. [80] analysed the effect of an educational visit to a dairy farm on the perceptions concerning farming practices of Canadian citizens with little prior knowledge of dairy farming. Most of the participants were unaware of the practice of early cow-calf separation, but raised concerns about it after the farm tour. A survey among Brazilian participants living in urban environments, with little or no association with dairy production, came to similar results [81]. In general, they were unaware that cows are separated from their calf at birth and most participants did not support this practice independent of provision of additional information.

In light of these findings the available scientific literature on the effect of early cow-calf separation on cow and calf health was systematically reviewed [82]. The authors concluded that the evidence extracted from the included journal articles does not support a recommendation of early dairy cow-calf separation on the basis of calf or cow health. The articles addressing calf diarrhoea pointed to beneficial or no effects of suckling. Mixed results were found for cryptosporidiosis, other common pathogenic causes for diarrhoea have not been evaluated in detail. Furthermore, no consistent evidence of increased risk of pneumonia in calves reared with the cow was found. For measures of calf immunity and mortality, no consistent pattern was observed, with studies split between those showing benefits versus risks associated with suckling. However, in studies detailing the risks of leaving the calf with the dam, colostrum intake was not systematically monitored. With respect to cow health, the review indicates that suckling is protective against mastitis and that cow-calf contact is not a significant risk factor for Johne’s disease. Early separation (within 24 h postpartum) was found to reduce acute distress responses of cows and calves in a review on the effects of prolonged cow-calf contact [83]. However, longer cow–calf contact typically had positive longer-term effects on calves, promoting more normal social behaviour, reducing abnormal behaviour, and sometimes reducing responses to stressors. Calf weight gains were increased during the milk-feeding period, when cows were allowed to nurse their calves, without consistent evidence of reduced milk production over a longer period.

Individual housing of dairy calves is another contentious practice very common on dairy farms [84]. European legislation allows calves to be kept individually for the first 8 weeks of life, however, it prohibits solid walls in individual calf pens, so that calves have to be able to have tactile contact to other calves (Council Directive 2008/119/EC). Individual housing of young calves is preferred by many producers, since it is perceived that group housing increases the risk of infectious disease, even though scientific evidence is ambiguous on this issue [5]. However, especially housing calves in larger groups requires more skills and poses more challenges to management [85]. Several studies have looked into the effects of housing calves in pairs under the assumption, that this smallest possible group would enable calves to socialize more naturally while minimising the disadvantages of group housing [86–90]. None of these studies found a negative impact on the health of calves housed in pairs. The advantages of pair housing are comparable to those of housing calves in larger groups, as reviewed by Costa et al. [91]. The reviewed studies showed that individually reared calves show deficient social skills, difficulties in coping with novel situations, and poorer learning abilities. Social housing for calves also improves solid feed intakes preweaning and helps improve weight gains before and after calves are weaned from milk to solid feed.

## Conclusions

Enhanced colostrum intake and a subsequent biologically normal (intensive) milk feeding programme support body growth and organ development in dairy calves. Only providing traditional restricted feeding is detrimental to resistance to disease, life-time performance and leaves calves hungry for long periods of time. This practice is therefore not consistent with animal welfare principles. Other contentious practices in the dairy industry, like early cow-calf separation and subsequent individual housing of the dairy calf, gain increasing attention from the general public. Scientific evidence does not support the common opinion, that these practices are beneficial for the health of calf or cow. Profound changes in current calf management practices are needed to improve dairy calf health and survival, enhance long-time performance of dairy heifers and satisfy consumer interests in farm animal welfare.

## Data Availability

Not applicable.
